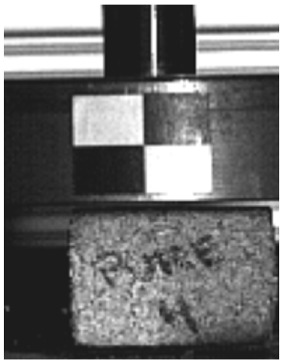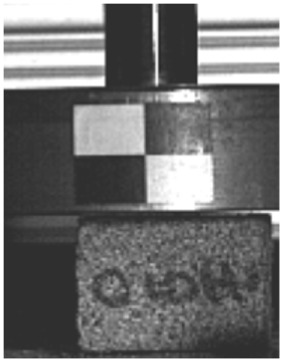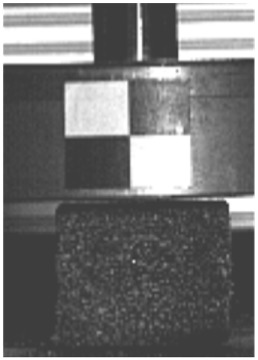# Graphene-Enriched Agglomerated Cork Material and Its Behaviour under Quasi-Static and Dynamic Loading

**DOI:** 10.3390/ma12010151

**Published:** 2019-01-04

**Authors:** Mariusz Ptak, Paweł Kaczyński, Johannes Wilhelm, José M. T. Margarido, Paula A. A. P. Marques, Susana C. Pinto, Ricardo J. Alves de Sousa, Fábio A. O. Fernandes

**Affiliations:** 1 Faculty of Mechanical Engineering, Wroclaw University of Science and Technology, Łukasiewicza 7/9, 50-371 Wrocław, Poland; mariusz.ptak@pwr.edu.pl (M.P.); pawel.kaczynski@pwr.edu.pl (P.K.); johannes.wilhelm@pwr.edu.pl (J.W.); 2 TEMA: Centre for Mechanical Technology and Automation, Department of Mechanical Engineering, University of Aveiro, Campus de Santiago, 3810-193 Aveiro, Portugal; jmtm@live.ua.pt (J.M.T.M.); paulam@ua.pt (P.A.A.P.M.); scpinto@ua.pt (S.C.P.); fabiofernandes@ua.pt (F.A.O.F.)

**Keywords:** cork, composites, graphene, mechanical tests, crashworthiness, energy-absorbing materials, natural materials

## Abstract

The use of cork for a variety of applications has been gaining significance due to environmental concerns and political agendas. Consequently, its range of applications is growing rapidly. In this work, aiming to improve its mechanical response for crashworthiness applications, cork agglomerates were enriched by small quantities of graphene oxide or graphene nanoplates in order to observe a resulting improvement of the mechanical behaviour during quasi-static and dynamic compressive loading cases. To produce homogenous cork agglomerates including graphene, the material was previously dispersed into granulated cork using stirrers to achieve a good distribution. Then, the typical procedure of compression and curing was carried out. Magnified images attest a good dispersion of graphene into the cork matrix. Mechanical testing was performed for a variety of graphene concentrations (0.1, 0.5 and 1.0 weight %), becoming clear that the beneficial effect of including graphene (either oxide or nanoplates) is related to a later densification stage while keeping the same stress plateau levels.

## 1. Introduction

There is increasing interest in cellular materials as a solution for many engineering applications. They have a huge potential that has been not fully exploited yet [1]. They offer a multitude of unique properties such as good thermal and acoustic insulation, high energy absorption capacity and high yield stress with respect to their mass [2]. Liu, et al. [3] and later Fu, et al. [4] devised a series of bio-inspired cellular structures based on bamboo and palm. They found that the specific energy absorption of the bionic design was superior to that of the single tube. Factors as these make cellular materials thought-provoking in many practical uses such as packaging, aerospace industry and many other structural applications [5,6].

One can state that the best material for each application depends on the application itself. Regarding crashworthiness, Expanded Polystyrene (EPS) is one of the most widespread synthetic cellular materials thanks to its excellent cost-benefits ratio. For this reason, most of protective headgear (from sports to roads) contains EPS. Nevertheless, this material is crushable, deforms plastically, hence offering minimal protection in multi-impact cases [7,8,9,10,11,12]. Therefore, other synthetic cellular materials with a viscoelastic behaviour are being introduced, such as Ethylene Vinyl Acetate (EVA) or Expanded Polypropylene (EPP). These materials provide good impact resistance under repeated loading [2,7], but at the expense of a less attractive market price.

As a common factor to all previously referred materials, the recyclability has been becoming a very serious issue. Oil-derived plastics are one of the major sources of world’s pollution [13,14] and it is necessary to start finding natural, sustainable alternatives to these materials.

Regarding crashworthiness, many researchers have been attracted by cork mainly due to its capability to absorb energy [15,16,17]. Cork comes from a not harmful outer bark extraction from *Quercus Suber* tree. The bark totally renews in approximately nine years. Agglomerated cork, a by-product mainly from wine-stoppers production can ally mechanical viscoelasticity to other well-known insulation properties of cork (thermal, acoustic, vibration), making it a quite interesting material from the engineering point of view.

Agglomerated cork is produced by compressing cork grains together (previously grinded to a specific grain size). A binder is added to keep the agglomerate structurally stable. Then, the compressed volume is cured in an oven. In the end, cork agglomerates are composite materials and their mechanical properties can be adjusted like any other composite by altering its density, percentage of binder, grain size or binder type, as demonstrated by Santos et al. [18] and Pereira [19,20].

Some authors have been proposing techniques and solutions to enhance the mechanical properties of cork agglomerates: Fernandes, et al. [21] showed that it is possible to increase both the elastic modulus and tensile strength by adding coconut fibres to the agglomerate, while Barnat-Hunek, et al. evaluated the physical and mechanical properties of heat-insulating mortars with expanded cork aggregates and different binders [22]. A comprehensive review on cork composites was carried out by Gil [23].

The innovative point and main objective of this study is to test and validate the use of graphene as a tool to enhance the mechanical behaviour of agglomerated cork under compressive loading. Indeed, given its outstanding properties, graphene composites are being introduced in a wide range of fields, from electronics and mechanics to medical fields. Graphene, the thinnest existing material, provides high thermal conductivity, outstanding mechanical features (Young’s modulus of 1 TPa and intrinsic mechanical strength of 130 GPa), high optical transmittance and high electronic transport [24,25]. Other comprehensive reviews on the new wave of graphene-based functional materials are given for instance in [26,27,28,29,30].

The authors’ study aims to add another remarkable application to graphene by dispersing graphene oxide or graphene nanoplates into the agglomerated cork matrix. Next sections will show the methods carried out and the resulting beneficial outcome in terms of mechanical properties.

## 2. Materials and Methods

The cork grains used in the experiment were provided by Amorim Cork Composites (ACC), with particle size of 0.5–1 mm. The graphene nanoplates (GNP) powder was provided by Cheap Tubes Inc. (Cambridge, UK) and used as given, while graphene oxide (GO) (4 mg/mL aqueous dispersion) was purchased from Graphenea (San Sebastián, Spain). GO was dried by lyophilisation and then submitted to a ball milling process in order to obtain small sized particles and gather uniform dispersion in the final composite.

The binder used in this experiment was supplied by Flexpur (Ovar, Portugal), and denominated as flexible due to its chemical and physical features. The binder is based on most common used isocyanates, which are the aromatic diisocyanates, toluene diisocyanate (TDI) and methylene diphenyl diisocyanate (MDI). In their work, Santos, et al. [18] also used Flexpur binders in the preparation of cork agglomerates. The authors investigated the chemical composition of the binders using a Fourier Transform Infrared Spectroscopy-Attenuated Total Reflectance, (FTIR-ATR) analysis, and the results indicated that, most probably, the flexible binder is a PU pre-polymer-based TDI.

**Figure 1:**
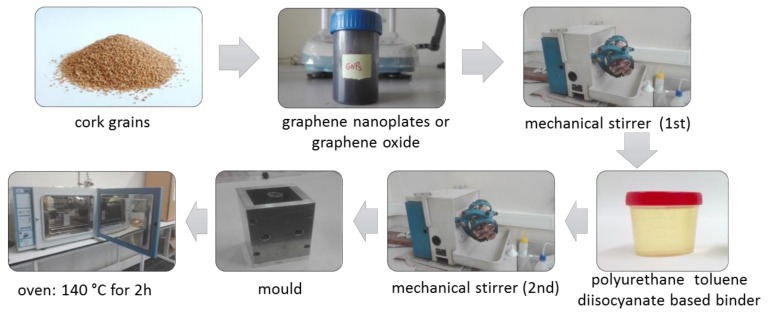
The cork-graphene samples production.

The preparation of the specimens commenced by the authors consists of four main steps. At first, it was necessary to weight the exact amount of constituents, which were necessary to obtain the assumed density of 160 kg/m^3^ in an analytical balance. It shall be noticed that the weight % (wt.%) is herein related to the total weight of the mixture—i.e., cork grains, binder, water and graphene. In the second step, the graphene nanoparticles or graphene oxide was added to the agglomerated cork. The components were mixed for 3 h. Hereafter, water (5 wt.%) was added to the cork part while the blend was kept homogenized using a mixer. One minute later, the binder (10 wt.%) was introduced to the mixture. In the last step, the mixture was compressed by a pressing machine until the desired density and shape was achieved. Furthermore, the samples were heated up to the temperature of 140 °C and held at that temperature for additional 2 h. The sequential production process of the cork-graphene samples is depicted in Figure 1.

**Figure 2:**
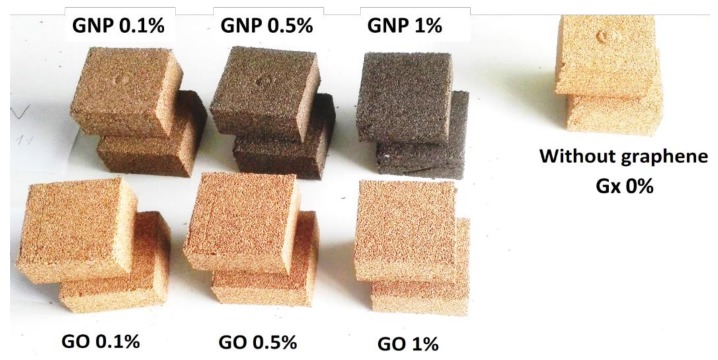
Example of samples subjected for quasi-static and dynamic testing.

Within the production process, the authors created three samples for each kind of composite, separately for both forms of testing quasi-static and dynamic. In total, the authors performed tests on 42 samples. The description and further used nomenclature is presented in Table 1. The overview of the produced samples of cubic shape (25 × 25 × 25 mm) is presented in Figure 2.

**Figure 3:**
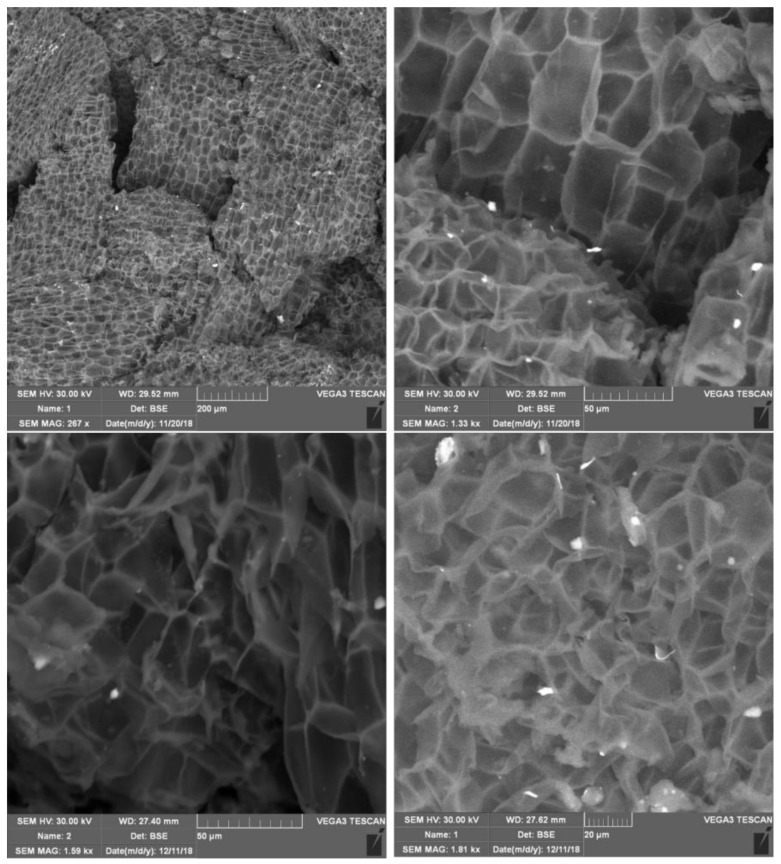
Microstructure of GNP sample with 1 wt.% graphene nanoplates: magnitude of 267 (**left**) and 1330 (**right**). Top row: specimen before test—magnitude of 267 (**left**) and 1330 (**right**), lower row: specimen after dynamic tests—magnitude of 1590 (**left**) and 1810 (**right**).

The samples were inspected by the use of a Scanning Electron Microscope VEGA3 (TESCAN, Brno, Czech Republic) in order to observe the distribution of the added graphene nanoplates or graphene oxide to the agglomerated cork material. Cork in its axial and tangential section is visible as brick wall and in its natural radial section mostly as heptagonal, hexagonal and pentagonal cells [31]. Exemplarily, the microstructure of an agglomerated cork sample with 1 wt.% of graphene nanoplates is shown in Figure 3. The average grain size was equal to 200 µm and the single cell of cork varied from 20–40 µm.

In the experiments, it is intended to observe the effect of GNP or GO presence and concentration on the agglomerated cork mechanical behaviour. In all tests, only the percentage of the reinforcement by GNP or GO was varied to the values of 0.1, 0.5 and 1 wt.%. As it was shown in Figure 2 control tests with agglomerate cork specimens without any reinforcement were also performed for comparison purposes. The other parameters, namely density, percentage of binding agent and the type of grain were kept constant throughout the entire time.

The experiments were carried out in two stages: quasi-static and dynamic loadings. The Shimadzu AG50 KN universal testing machine (Shimadzu Scientific Instruments, Kyoto, Japan) was used for the quasi-static compression tests. Thereby, the samples were subjected during the test to compression at a strain rate of 0.1 s^−1^. The recorded load-deflection-curves were used in order to calculate the stress-strain.

The dynamic tests were performed by using the Instron Dynatup 9250hv (Instron, Norwood, MA, USA). The dynamic tests were separated into first and second impact for each specimen. As the tests were carried out in a row on the same day, the authors ensure constant temperature conditions for all tests. The samples were impacted by a 7.05 kg flat tup with a 50 mm diameter, which is initially positioned 300 mm above the specimen top surface. Hence, the applied kinetic energy was set to 20.75 J. The tests were scheduled in the way that after a relaxation time of 10 min, the second impact was carried out for the same specimen. For the second impact, the height of the tup was adjusted for each specimen separately to 300 mm, guaranteeing the same impact energy of 20.75 J. Data acquisition for the dynamic tests was done by the use of a load cell with a sample rate of 204.8 Hz. Additionally, a high-speed camera Phantom V12 (Vision Research, Wayne, NJ, USA) was used at a frame rate of 10,000 fps with an exposure time of 40–90 μs. The gathered video-files were processed by using TEMA Motion (Image Systems Motion Analysis, Linköping, Sweden). The dedicated software allowed the authors to generate for each specimen accurate displacement-time data for characteristic, beforehand installed high-contrast markers. These markers were positioned on the machine’s impactor and anvil. As the sample rate was differing for load cell data and video data, the data correlation was done by the use of National Instruments LabVIEW 2014 software.

## 3. Results and Discussion

### 3.1. Quasi-Static Tests

**Figure 4:**
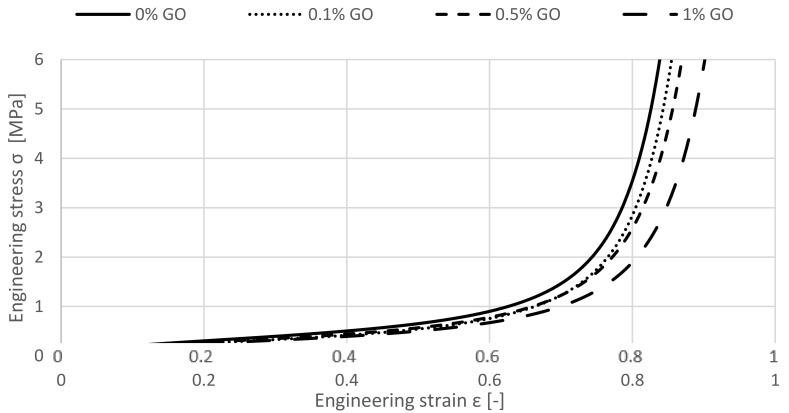
The influence of the GO used as a reinforcement on uniaxial quasi-static compression.

The agglomerated cork samples and the cork samples enriched by Graphene Oxide exhibit firstly a comparable performance under quasi-static compression. By observing Figure 4, it can be recognized that this behaviour ends approximately at a strain of 0.4 when the specimen with higher amounts of GO starts to develop a longer but not higher plateau stage.

Consequently, densification establishes later. This is a desirable feature of energy absorbing materials, deforming at almost constant stress values and achieving densification for high compressive strains. Thus, a gradual enhancement of the crashworthiness properties can be recognized by increasing the wt.% of GO to the agglomerated cork specimen. According to this observation, the most significant changes majorly occur from 0 to 0.1 wt.% and from 0.5 to 1 GO wt.%.

**Figure 5:**
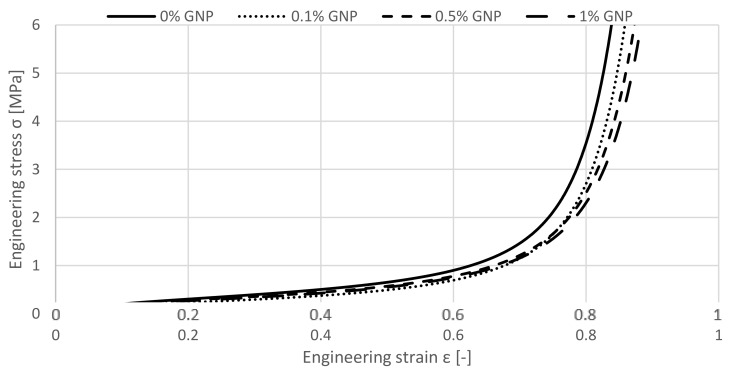
Influence of the GPNs used as a reinforcement on uniaxial quasi-static compression.

Figure 5 shows the results of the GNP-enriched specimen. It is visible that the specimens present a similar behaviour compared to the GO-enriched specimen. Up to a strain of approximately 0.4, the curves are close to each other. After surpassing the total strain of 0.4, an elongated plateau stress is also recognizable. Here, the most visible beneficial effect is seen for a change from 0 to 0.1 wt.%.

In appropriate applications of energy absorption, the plateau effect is the most relevant segment of the stress-strain curve. The longer the plateau phase, the better applicability of the solution can be stated. From this particular point of view, the GNP and GO enriched specimens are interesting, once they lasted longer showing such behaviour, with material reaching densification for more than 80% uniaxial compression. On the other hand, it needs to be noted that the plateaus stress values were not increased. Therefore, by remaining stable in terms of stress values, but with a delayed densification, it can be concluded that graphene promotes a beneficial effect in terms of mechanical properties. Consequently, the attractiveness of adding this nanomaterial is raised for any kind of composite as a reinforcement.

**Figure 6:**
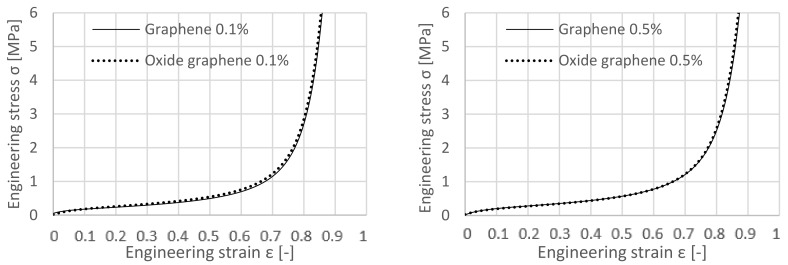
Comparison between samples with 0.1 wt.% GNP and 0.1 wt.% GO (**left**), Comparison between samples with 0.5 wt.% GNP and 0.5 wt.% GO respectively (**right**).

Comparing the results of equally GNP- and GO-enriched specimens with each other, Figure 6 shows very similar performances of the specimens that were enriched by 0.1 and 0.5 wt.% of GO and GNP, respectively. Their stress-strain curves are practically coincident.

**Figure 7:**
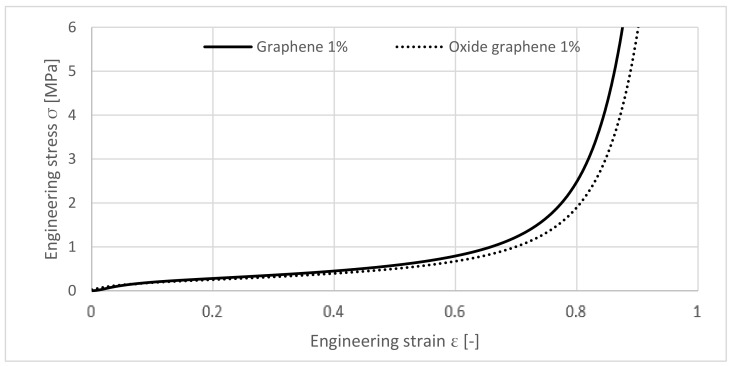
Comparison between samples with 1 wt.% GNP and 1 wt.% GO.

By comparing the added quantity of 1 wt.% of GO or GNP to the specimen in Figure 7, their behaviour is characteristically in several aspects. The GNP sample shows a higher plateau but densifies first.

Adding 1 wt.% GNP has better effects on the improvement of stress-strain curves compared with GO, namely an elongated plateau stage. Such a difference is mainly related to the chemical nature of these two fillers. GO sheets owns oxygen functional groups at their surface, making it a polar nanomaterial. On the contrary, GNPs are only made by carbon (apolar nanomaterial). The cork grains are also apolar. Relating this to the mixing procedure within the production process, it is more likely to obtain a better dispersion of the GNP on the cork grain, yielding in better results.

One must however bear in mind, that in both types of reinforcement their most significant changes in performance were due to higher quantities of added GO or GNP, higher than 0.5 wt.%. Above 1 wt.% a saturation of the material is expectable with no further improvements.

### 3.2. Dynamic Tests

The authors were especially interested in the influence of adding graphene oxide or graphene nanoparticles to agglomerated corks mechanical properties—particularly stiffness—under dynamic loading. The benefit would be seen in a material with high energy absorption capabilities, which is not about to fail suddenly by reaching higher stress levels. Table 2 illustrates the observed compression process for the used composites types, which were subjected to 20.75 J impacts with a flat tup.

**Figure 8:**
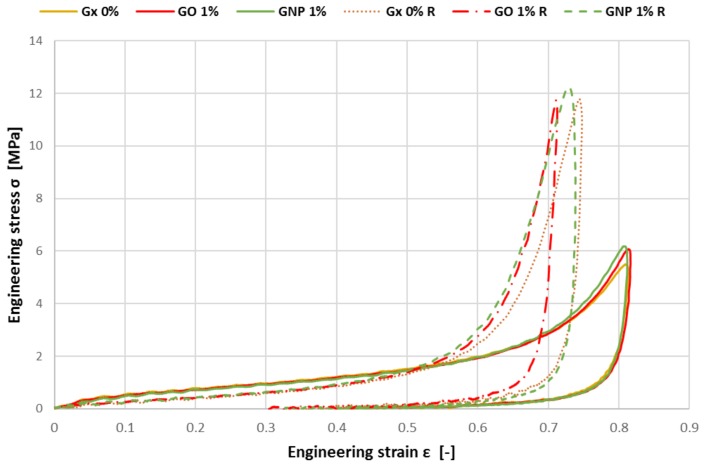
Stress-strain curves for the specimens during the first impact and second impact (marked by R).

Figure 8 shows the stress-strain curve for the first impact. It can be seen that the curves are not establishing different plateaus as in the quasi-static tests, even not by enriching cork with 1 wt.% of GNP or GO. The total absorbed energy was calculated for each specimen—the results showed no significant change. The only tendency can be seen in the slightly increasing stress values for the same strain, when GO or GNP were added.

**Figure 9:**
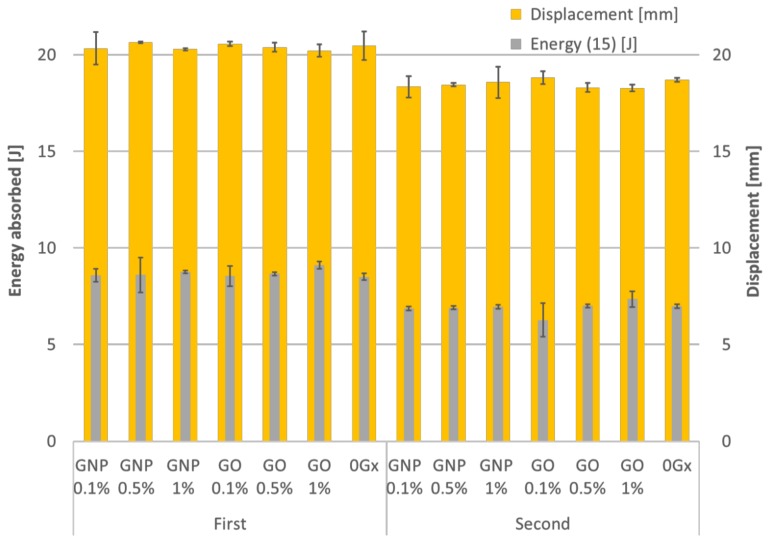
Absorbed energy (Energy(15)) at a sample deflection of 15 mm and displacement chart for the first and second impacts.

As cork is generally accepted as a material capable to withstand at least a second major impact, each specimen was tested for the second time in the same conditions. The behaviour of all the different samples is very similar. After analysing the results, the dynamic performance of GO- or GNP-enriched cork material can be stated as very comparable to each other, showing no significant difference, neither to the other type of enrichment nor to the untreated agglomerated cork. Figure 9 shows the overview of absorbed energy and displacement for the first and second impacts for each material configuration. For comparison purposes, a deflection threshold of 15 mm—marked as Energy(15)—was established. Such a value was set once all samples were able to withstand impacts at this stage.

In this work, the authors compared the quasi-static versus dynamic response of the graphene-enriched agglomerated cork material. We noticed that unlike for the quasi-static loading, during the dynamic tests, enriching samples by graphene has no significant influence on the energy absorption capabilities compared to the cork agglomerate without added graphene. Since the data was verified, and the tests were carried out on three samples for each test, the experiment is considered to be robust. Therefore, the explanation of this interesting phenomenon is related to the binder (polyurethane glue), impact energy and the binder reinforcement i.e., GNP or GO. First of all the cork material, in comparison to the used polyurethane binder, is a very good heat insulator with small heat capacity [32,33]. Once, during the dynamic test, a sample is impacted by the tup at the kinetic energy of 20.75 J, there are thermal effects observed in the samples—mainly due to its viscoelastic and viscoplastic nature of cork agglomerates. The compression of the air within a cork sample and its cells occurs so rapidly that on the time scale of the compression process, little of the system energy can be transferred out as heat. Thus, the dynamic impact can be regarded as an adiabatic process. Since the cork grains have small heat capacity, the cork grains heat the glue, which binds the cork grains. The added thermal energy to the system causes the glue under the compression to have less adhesive capabilities compared to the samples at quasi-static test at 21 °C. Therefore, we quasi-static test can be seen as isothermal. This is because of the fact, that the change in the system occurs slowly enough, which allows the glue to continue to adjust to the temperature of the cork sample through heat exchange. In a consequence, all these effects in dynamic tests make the graphene oxide or graphene nanoplates to lose their importance as the cork-agglomerate reinforcement—as observed for quasi-static tests. In other words, once the viscosity of binder decreases and it has less adhesive compared to the binder in quasi-static tests, the integrated-in-the-binder GNP or GO reinforcement is not influencing the energy-absorbing capabilities. This is clearly visible in Figure 9 where GNP or GO does not increase the crashworthiness of graphene-enriched samples compared to the 0Gx benchmark samples (no graphene added)—neither for first nor for second impact test. Overall, as the phenomenon has not been explained in the literature yet, the authors believe it may be an initiation for further research is the field of natural material crashworthiness.

## 4. Conclusions

This research was intended to create a reinforced agglomerated cork material. Therefore, graphene nanoplates or graphene oxide was added in the quantities of 0.1, 0.5 or 1 wt.%. Graphene holds promise for various material and device applications due to its exceptional mechanical properties. Nevertheless, in numerous works and research it was a struggle to blend it homogenously into cork with success. The presented work contributes to this field by creating original composites enriched by GNP or GO. The samples were observed concerning the distribution of the added GNP or GO by the usage of SEM and consecutively tested toward their mechanical response to quasi-static and dynamic loads. The authors conclude, that:

▪ Adding GNP or GO to agglomerated cork material extends the plateau stress and delays densification under quasi-static loads.
▪ The most significant changes in these effects were recognized for the change from 0 to 0.1 wt.% and 0.5 to 1 wt.% of added GO or GNP.
▪ In contrast to quasi-static loading, during the dynamic tests, for the particular testing conditions (impact energy and impact velocity) enriching samples with graphene has no significant influence on the energy absorption capabilities compared to the non-reinforced cork agglomerate.
▪ Enriching samples by GNP or GO in the tested quantities showed limited influence on the energy absorption capabilities, that is, its effect is only evident for low strain rates. Nevertheless, in the latter, it clearly delayed densification stages, promoting better crashworthiness properties. Damage resistance was also not found to be enhanced.

Overall, the findings of this research contribute to possible applications of this reinforced cork material. The revealed effect of extending the plateau stress gains importance in applications exemplarily as damper of heavy-load machinery, particularly, when weight is a limitation. Thereby, the findings are opening a new material resource.

## Figures and Tables

**Table 1:** Description and nomenclature used for the produced samples.

Description	Nomenclature Used
cork with 0.1% of graphene oxide	GO 0.1%
cork with 0.5% of graphene oxide	GO 0.5%
cork with 1% of graphene oxide	GO 1%
cork with 0.1% of graphene nanoparticles	GNP 0.1%
cork with 0.5% of graphene nanoparticles	GNP 0.5%
cork with 1% of graphene nanoparticles	GNP 1%
without graphene	Gx 0%

**Table 2:** Time sequence of the first impact for 3 different composite types.

Type/Time	0Gx	GO1%	GNP1%
**1.0 ms**	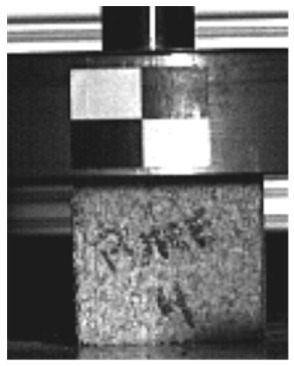	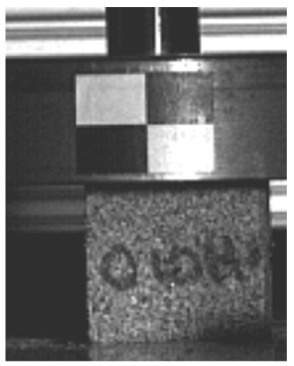	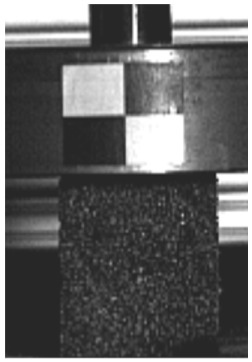
**4.0 ms**	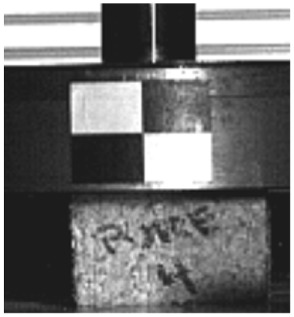	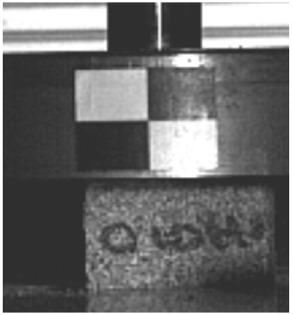	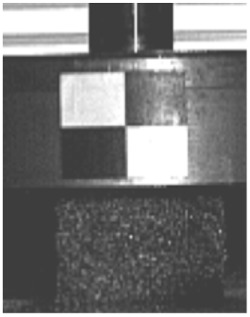
**7.0 ms**	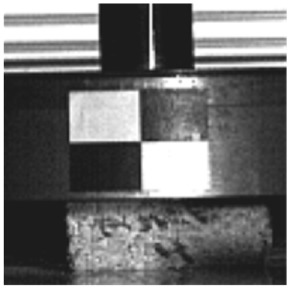	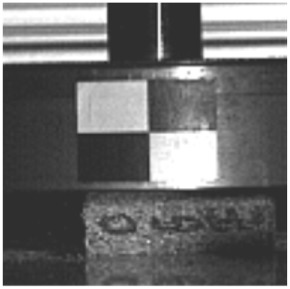	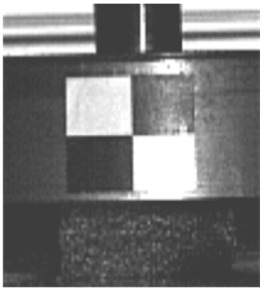
**10.0 ms**	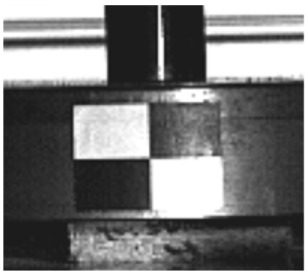	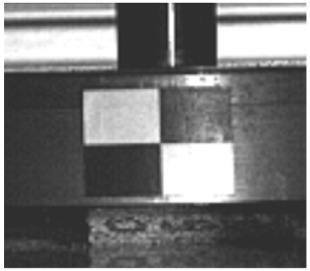	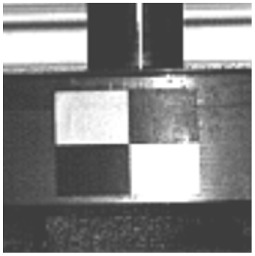
**30.0 ms**